# Anti-Aging Effects of a Serum Based on Coconut Oil Combined with Deer Antler Stem Cell Extract on a Mouse Model of Skin Aging

**DOI:** 10.3390/cells11040597

**Published:** 2022-02-09

**Authors:** Truc Le-Buu Pham, Thuy Truong Thi, Huyen Thi-Thuong Nguyen, Thuan Duc Lao, Nguyen Trong Binh, Quan Dang Nguyen

**Affiliations:** 1 Department of Animal Biotechnology, Biotechnology Center of Ho Chi Minh City, Ho Chi Minh City 700000, Vietnam; truongthithuysps2209@gmail.com (T.T.T.); ntbinh.snn@tphcm.gov.vn (N.T.B.); 2 Faculty of Biotechnology, Ho Chi Minh City Open University, Ho Chi Minh City 700000, Vietnam; thuan.ld@ou.edu.vn; 3 Department of Biology, Ho Chi Minh City University of Education, Ho Chi Minh City 700000, Vietnam; huyenntth@hcmue.edu.vn

**Keywords:** deer antler stem cells, mouse, ultraviolet A (UVA), virgin coconut oil (VCO)

## Abstract

Anti-aging is one of the top goals in the field of health care and aesthetics. Anti-aging cosmetics derived from nature are oriented to long-term development, bringing safety to users and being environmentally friendly. The aim of this study was to develop an anti-aging cosmetic formulation process based on coconut oil in combination with deer antler stem cell extract. The results show that the presence of deer antler stem cell extract added to the foundation made the serum product highly stable and helped improve skin aging significantly after 2 weeks of use. The skin site where the serum product was applied showed a smooth and elastic skin surface, with very few fine lines and shallow wrinkles. Serum reduced the number of wrinkles (48.09% compared to commercial serum (ME) and 60.31% compared to positive control (PC)), reduced skin recovery time (39.31% compared to ME and 67.1% of PC) after two weeks of use. After 2 weeks of use, collagen density increased 10.18% compared to ME and 63.76% compared to control. Epidermal thickness increased by 106.1% compared to PC and 121.7% compared to ME.

## 1. Introduction

The skin is the largest organ of the human body and serves as a protective barrier against biological, chemical and physical stress. Skin aging is a visible signal and one of the representative signals of aging changes [1]. 

The main external signs of skin aging include the appearance and increase of wrinkles and loss of skin density [2,3]. Aging inside the skin is a long and slow process. When the aging process takes place, the epidermis shows signs of rapid keratinization, slow cell regeneration, increased sensitivity to UV rays, making the internal structure of the skin weak and susceptible to damage, infections and slow wound healing [2]. The aging of the dermis is manifested through the deterioration of connective tissue, bundles of connective fibers, broken capillaries, poor blood circulation leading to a decrease in the efficiency of nutrient transport and oxygen to the surface of the skin. Along with the lack of elastin and the loss of a large amount of natural collagen, the dermis tissue is destroyed, the skin structure becomes weaker. This is considered the main cause of wrinkles on the skin, reduced skin density and reduced elasticity of the skin [2].

There are two main causes of skin aging: intrinsic factors and extrinsic factors. Changes in individual genetic background are thought to govern the intrinsic and time-dependent aging process [4]. Ultraviolet (UV) radiation is an extrinsic factor capable of premature skin aging and is considered to be responsible for 80% of facial aging [5], affects all 3 layers of the skin, in which the epidermis and dermis are affected and have the most pronounced changes [6]. Damaged skin from UV exposure is characterized by an increase in the number of dead skin cells, reactive oxygen species (ROS) as well as the dimers thymine and pyrimidine, which produce collagenase and induce an inflammatory response [7]. ROS species activate matrix metalloproteinases (MMPs) involved in collagen damage, disrupting extracellular matrix-binding proteins leading to structural disruption of the skin, causing sagging and wrinkling [8]. Anti-aging and anti-aging cosmetics are a matter of great interest to many people. The purpose of anti-aging cosmetics is to stimulate the regeneration of physiological processes inside the body to help improve skin aging while protecting the skin from aging agents [9]. These cosmetic products specifically contain ingredients or nutrients that help promote healthy skin and may include vitamins (C, E), minerals, and antioxidants (kinetin, lipoic acid and compounds). Chemicals such as polyphenols, flavonoids, arbutin and carotenoids [10,11,12,13]. Safe, natural products are gaining attention, and plant compounds are increasingly favored as cosmetic ingredients because they can protect and heal the skin [14]. Essential fatty acids and triglycerides containing unsaturated fatty acids are the foundation of cosmetic product development and formulation. Because of their integration in cell membranes and restoring the damaged lipid barrier of the epidermis and preventing water loss, oils with a high content of linoleic and α-linolenic acids are important in preventing disease atopic dermatitis and is used as an integral component of cosmetic preparations [15,16].

Coconut oil is a potential candidate for natural skin care products with properties such as antibacterial, anti-inflammatory and moisturizing. Virgin Coconut Oil (VCO) consists of 90–95% saturated triglycerides that have a moisturizing effect on the skin [17]. VCO contains high levels of ferulic acid and p-coumaric acid (both phenolic acids) which are associated with antioxidant capacity [18]. Clinical studies show that coconut oil improves symptoms of skin disorders by moisturizing and soothing the skin [19]. In a study of the anti-inflammatory and skin-protective properties in vitro of VCO showed moderate UV resistance in HaCaT cells. VCO exerts its anti-inflammatory activity through suppressing inflammatory markers and protects the skin by strengthening the skin barrier function [19]. Another study showed 100.36% improvement in skin moisture content of mice after 2 weeks of using VCO and 148.89% after 4 weeks compared with before application. Compared with before application, the rate of water loss through the epidermis improved by 27.70% 2 weeks and 36.97% after 4 weeks of applying VCO [20]. Evidence suggests that VCO has the ability to promote wound healing through the promotion of epithelial regeneration and collagen synthesis [21].

Skin care products containing active ingredients that are hydrophilic or hydrophobic; each has its limitations when used in topical formulations. In order to overcome these limitations, it is necessary to consider many factors such as the skin on the human body, the duration of use, the mechanism of action and possible side effects [22]. The potential of microemulsions has been of great interest because of its stability, ease of preparation, low viscosity and high solubility for different types of substances including hydrophilic and lipophilic molecules and at the same time enhance product stability and prevent irritation [23,24]. 

A study that used VCO microemulsions with surfactants to fill gaps in biomedical materials demonstrated the positive effects of VCO such as antibacterial effects, wound closure efficiency 95 ± 2% on day 14 and significantly higher than commercial products and materials without VCO addition (91 ± 4%) [25].

Stem cells and secretions are of interest, a 2020 study by Kim et al on cosmetics containing human stem cell conditioned medium (ADSC-CM) showed positive effects : increase moisture, reduce transepidermal water loss (TEWL), smooth wrinkles and whiten skin [26].

In 2015, Son et al. performed a study to examine the effects of adipose-derived mesenchymal stem cells (AdMSCs) in UV-irradiated human skin fibroblasts (HDFs) on their therapeutic potential in reducing wrinkles.The study determined that AdMSC decreased MMP-1 levels in UV-irradiated HDF and increased AdMSC dose-dependent procollagen type 1. This result provides evidence of a relationship between MMP-1 and procollagen production to protect against wrinkle formation [27]. 

Recently, active substances secreted from deer antler stem cells have been used in regenerative medicine as cytokines and growth factors that play an important role in affecting the microenvironment of the host tissue. Deer antler stem cells exert paracrine effects through the secretion of 26 growth factors. In particular, PDGF, VEGF and TGF-b2 are abundantly secreted from deer antler stem cells, promoting cell migration and increasing the wound healing ability of fibroblasts in vitro and in vivo [28].

Currently, studies on active ingredients extracted from deer antler antler are very few and the potential for exploitation from deer antler stem cell culture is very large. Deer antler stem cells have shown great potential for anti-aging skin. With the method of emulsifying coconut oil by emulsifying agent and adding active ingredients extracted from deer antler velvet, it is possible to promote the advantages of VCO while limiting the side effects. In order to have more scientific basis for the effective exploitation of cell sources in the field of cosmetology, we conducted a study on the preparation of Serum based on coconut oil combining with deer antler stem cell extract and evaluate the anti-aging effect of the product on the mouse model of skin aging.

## 2. Materials and Methods

### 2.1. Approved by the Animal 

This research work has been approved by the of the Animal care and use committee of University of science, VNUHCM (ACUCUS) no 1170B/KHTN-ACUCUS date 11 November 2019.

### 2.2. Vietnamese Sika Deer (Cervus nippon pseudaxis)-Derived Stem Cells Extract Preparation

The deer antler stem cell extract was obtained from the culture condition medium without FBS of pass 5 deer antler stem cells. The extract was then quantified the levels of FGF2 and EGF by ELISA kit (ab99979, and ab217772) to check the expression of these target proteins. The extract was stored at 4 °C for further preparation experiments.

### 2.3. Prepare Serum from Coconut Oil Base Combined with Deer Antler Stem Cell Extract

Building phase diagram by titration method [29,30], water was added slowly to the mixture of oil, oil-phase emulsifier, water-phase emulsifier until a complete emulsion was obtained.

From the ratio of phases in the emulsifying system, formulating a complete coconut oil base cream with other active ingredients suitable for the characteristics of each phase and ensuring the stability of the system [31,32].

### 2.4. Animal Models

Animals used for the experiment were female mice weighing 19–21 g, marked with the treatments and mince in each treatment according to the code, and then shaved the day before the experiment.

The shaved area on the back has a minimum size of 9 cm^2^, after shaving, the skin needs to recover 6–12 h before UVA irradiation.

Top-mounted UVA lamp, 30 cm from the back of the mouse [33] with a capacity of 30 W, shines 6 h/day, 7 days/week, for 8 weeks continuously. After 8 weeks of exposure (W0), apply the product corresponding to each treatment (DD, ME, SR).

The experimental model was built with 5 groups of 9 mice each:

Group 1: Mice were raised under normal conditions under room light (Positive control—PC);

Group 2: Mice were exposed to UVA for 8 weeks and applied with distilled water (Negative control—NC);

Group 3: Mice were exposed to UVA for 8 weeks and applied a foundation made from coconut oil (Oil base—OB);

Group 4: Mice were exposed to UVA for 8 weeks and applied a commercial products with ingredients extracted from Deer Antler Velvet in Vietnam (ME);

Group 5: Mice were exposed to UVA for 8 weeks and applied experimental products (SR).

### 2.5. Wrinkle Quantity Assessment Method

Mince were anesthetized with Diethyl Ether via inhalation, after anesthesia, mice were naturally placed under a 9 cm^2^ blank square to determine the wrinkle assessment area. The number of individual wrinkles was counted in 3 repetitions and averaged over a defined area.

### 2.6. Methods to Assess the Recovery Time of the Skin

Evaluation of recovery after skin tightening was performed according to Bryce et al. (1991) and revised [34,35]. After the mouse is anesthetized, use your thumb and index finger to hold the dorsal skin between the vertebrae, lift until the mouse’s hind legs leave the table, then release your hand immediately and press the clock to determine. The time the back skin returns to its original state.

### 2.7. Method of Collecting Mouse Skin Samples and Staining with Trichrome

After 8 weeks of irradiation (week 0–0 W), after 1 week (1 W) and 2 weeks (2 W) of applying the product, the mouse was operated on.

Skin samples were collected, soaked in 10% formalin and stained with Trichrome (3 mouse/group).

### 2.8. Methods to Assess Epidermal Thickness

Random measurements were made at 10 sites on a slice of the skin sample using the S-EYE software. For each skin sample, measure 3 slices, repeat 3 times.

### 2.9. Methods of Assessing Collagen Density

The percentage of collagen was quantified on images of Trichrome-stained skin tissue samples using a NIS-Elements F 4.60.00 40× objective microscope and processed on ImageJ software [36].

### 2.10. Statistical Analysis

All data obtained from the study were statistically processed using Minitab 18: one-way analysis of variance (One-way Anova). The mean data are presented as $\bar{X}$ ± 95% CI. The level of significance used to test the significant difference of the treatments is 0.05 (*p*-value < 0.05, the difference is statistically significant).

## 3. Results

### 3.1. FGF2 and EGF Concentrations in Deer Antler Stem Cell Extract

The deer antler stem cell extract was obtained and the concentrations of FGF2 and EGF presented in the extract were analyzed by FGF2 and EGF ELISA Kit. The results indicated that FGF2 concentrate was 1.851 pg/mL and EGF concentrate was 87.864 pg/mL.

### 3.2. Prepare Serum from Coconut Oil Base Bombined with Deer Antler Stem Cell Extract

After the survey found that the formula with HLB = 5.5 for the most stability and sustainability, the HLB value of coconut oil was 5.5.

The recommended formula for the experiment is with an oil phase ratio (coconut oil and oil-soluble substances) about 60–70%, a mixture of emulsifiers 20–30% and a water phase (water, water-soluble substances, etc.). TBG extract) about 10–20%. The complete serum formulation containing 0.01–2.5% deer antler stem cell extract was evaluated in animal models.

### 3.3. Skin Appearance Characteristics 

**Figure 1:**
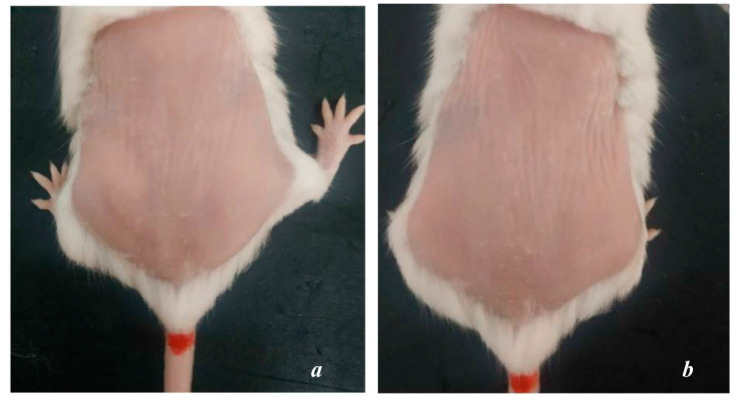
Mouse skin after 8 weeks of UVA exposure: ((**a**)—PC; (**b**)—NC).(**a**)The PC group showed many statistically significant superficial wrinkles.(**b**) NC group dry skin, uneven skin tone, deep wrinkles and a lot.

**Figure 2:**
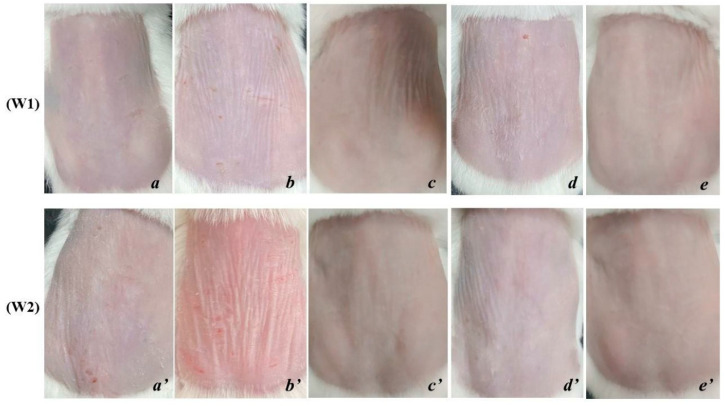
Mouse skin after 1 and 2 weeks of applying the experimental product (W1—after 1 week; W2—after 2 weeks; (**a**,**a’**)—PC; (**b**,**b’**)—NC; (**c**,**c’**)—OB; (**d**,**d’**)—ME; (**e**,**e’**)—SR). (**a**) PC, wrinkles are more and more obvious, (**a’**) wrinkles are more and deeper. (**b**) NC, many and deep wrinkles; (**b’**); dry, vulnerable skin, many and deep wrinkles. (**c**) OB, smooth, even skin tone, few wrinkles but deep; (**c’**)—smooth skin, few and shallow wrinkles. (**d**) ME, dry skin, many and shallow wrinkles; (**d’**)—few wrinkles, shallow, smooth skin. (**e**) SR, skin color is relatively uniform, smooth and with few wrinkles; (**e’**)—smooth skin, few or no wrinkles.

After 8 weeks of UVA exposure, group NC showed signs of aging such as dry skin, flaky epidermis, thin skin surface, high number of wrinkles, and vulnerability. The elasticity is poor, the skin takes longer to return to its original state (Figure 1b). The condition becomes more severe after 1 and 2 weeks of cessation of exposure, the skin is markedly thinned, wrinkled and less elastic (Figure 2b,b’). Meanwhile, the skin surface of PC mice is relatively smooth, with little or no wrinkles (Figure 1a).

In the OB group, the skin surface becomes thicker, the number of wrinkles is reduced and shallower. The skin surface is tight and the skin’s ability to recover is also improved compared to before applying the product and is different from PC and NC (Figure 2c,c’).

In the ME group, the skin tends to be smooth, and the elasticity is significantly improved. However, the surface of the skin is dry and vulnerable, the skin tends to thin, which makes the wrinkles on the skin still quite clearly present on the skin (Figure 2d,d’).

The skin of rats applied with SR showed a positive skin recovery effect and was completely different from the rest of the treatments. The skin surface is smooth, the skin’s resilience is fast, the number of wrinkles is significantly reduced, even wrinkles fade and are difficult to see, the protection ability of the skin is better (Figure 2e,e’). 

This shows that the effectiveness of the serum from deer antler stem cell extract significantly improves the external structural characteristics of the skin under the influence of UVA.

### 3.4. The Number of Wrinkles on the Skin 

**Figure 3:**
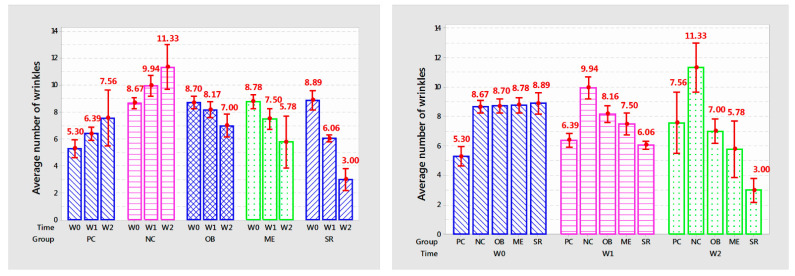
The graph shows the average number of wrinkles after each week of product application.

Statistical results on the number of mouse skin wrinkles are shown in Table 1 and Figure 3.

The number of mouse skin wrinkles after one and two weeks of applying the product in each group is different with very high confidence (*p* < 0.001). 

The number of wrinkles in the NC group increased rapidly and was different from that in the PC group (9.94 versus 6.39 after 1 week of application and 7.56 compared with 11.33 after 2 weeks, *p* < 0.001) (Table 1).

In the OB group, the number of wrinkles decreased and was different after 2 weeks of use (from 8.17 to 7.00, *p* < 0.05) compared to before treatment but still higher than the PC group at the same time point. survey time (8.17 versus 6.39; 7.00 versus 7.56, *p* < 0.001; respectively); in the ME group, the number of wrinkles decreased after each week of application (8.70 to 7.50 and 5.78 wrinkles, *p* < 0.001) but was not different from that of OB application after one week of application (*p* > 0.05); in the SR group, the number of wrinkles significantly decreased after each week (from 8.89 to 6.06 and 3.00 wrinkles, *p* < 0.001).

The overall effect showed that SR application reduced wrinkles quickly and was different from the other 2 topical products (SR decreased 5.1% compared to PC and 19.2% compared to ME after 1 week and 60.21% compared to PC), 48.09% compared to ME after 2 weeks of application).

In summary, with the obtained results, the serum from deer antler stem cell extract initially showed the effectiveness in anti-aging rat skin under the influence of UVA through reducing the number of wrinkles after 2 weeks of use.

### 3.5. The Ability to Recover Skin 

**Figure 4:**
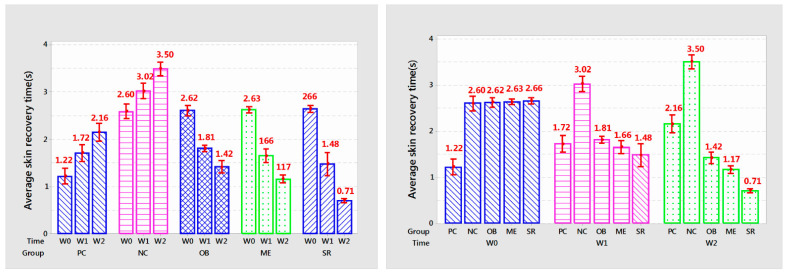
The graph shows the average number of skin recovery times after each week of applying the product.

Statistical results of mouse skin recovery time are shown in Table 2 and Figure 4.

After each week of applying the product, the recovery time of the rat’s skin in each group had significant and significant changes (*p* < 0.005). Skin recovery time of PC and NC treatments increased gradually over each week of experiment (*p* < 0.001). PC increased from 1.22 to 1.72 and 2.16 s (*p* < 0.01) every week and NC was 2.60; 3.02 and 3.50 s (*p* < 0.001).

OB reduced skin recovery time from 2.62 s to 1.81 and 1.36 s (*p* < 0.001); ME decreased from 2.63 s to 1.66 and 1.36 s (*p* < 0.001); SR showed the fastest reduction in recovery time from 2.66 s to 1.48 and 0.71 s after each week of treatment. Overall, the SR group showed the highest efficiency, helping to reduce 44.36–73.3% compared to before use and from 13.95–67.1% of the time compared to PC at the same time while ME only decreased from 36.88–55% compared to the original and 3.48–45% compared to PC.

### 3.6. Trichrome Dyed Skin Texture

**Figure 5:**
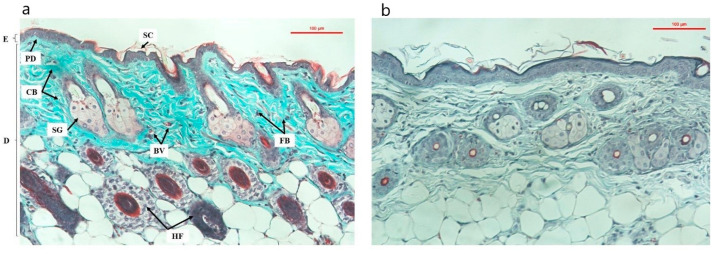
Mouse skin microstructure after 8 weeks of UVA (X20) irradiation ((**a**)—PC; (**b**)—NC). E: epidermis; D: dermis; PD: papillary dermis ; CB: collagen bundles; SG: sebaceus glance; SC: stratum corneum; BV: blood vessels ; HF: hair folicle; FB: fibroplast [37].

The structural PC group is relatively complete with stratification of the epidermis and the epidermis is thinner than that of the NC group. The dermis is closely connected with the upper epidermis, and the number of keratinized cells is small. The collagen structure is uniform and continuous, the collagen fibers are intact, not broken, the elastic fibers are arranged and branched, and the fiber bundles are tightly connected. Hair follicles and sebaceus glance were intact, attached to the hair follicles, and developed normally (Figure 5a).

The skin structure in the NC group appeared characteristic features of aging skin, epidermal thickening and hyperkeratinization, loose collagen structure, broken collagen fibers, discrete fiber bundles, loss of alignment, twisted and disorganized. The hair follicles, glands and blood vessels showed signs of stopping growth or regenerating more slowly than in the PC group, the cell system was less and more sparse than in the PC group (Figure 5b).

**Figure 6:**
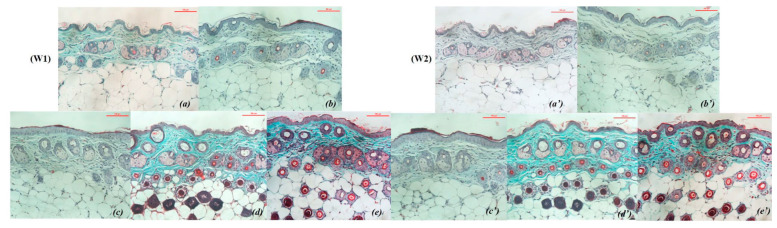
Trichrome-stained mouse skin structure after 2 weeks of treatment (X20): (W1—after 1 week; W2—after 2 weeks; (**a**,**a’**)—PC; (**b**,**b’**)—NC; (**c**,**c’**)—OB; (**d**,**d’**)—ME; (**e**,**e’**)—SR). (**a**,**a’**)—PC, very little difference, relatively similar to the time before applying the product. (**b**,**b’**)—NC, discrete fiber bundles, almost no collagen association, low collagen density. (**c**,**c’**)—OB, collagen structure is more interconnected, cell proliferation and a small amount of new blood vessels, epidermis still increases in thickness and tends to be relatively keratinized. (**d**,**d’**)—ME, relatively high collagen density, appearance of new cells and blood vessels under the skin. (**e**,**e’**)—SR, dense, connective collagen density, high number of secretory cells, hair follicle cells and new blood vessels.

In the groups that used the product (OB, ME and SR), the overall trend was for improved skin texture and reduced signs of aging. In the OB group, the collagen structure was more coherent, the density was higher than that at 8 weeks after UVA irradiation (Figure 5b), besides the proliferation of cells and a small amount of new blood vessels (Figure 6c,c’). However, the epidermis still increases in thickness and tends to be relatively keratinized, which is considered an undesirable effect from virgin coconut oil when taking care of the skin. After one and two weeks of applying the product, the ME and SR groups showed significant improvements in skin texture, relatively high collagen density, and an increase in secretory cells, new cells forming, increased blood vessels under the skin (Figure 6d,d’,e,e’, respectively). In particular, the SR treatment showed a higher effect with the following characteristics: higher collagen density, more secretory cells, hair follicle cells and new blood vessels (Figure 6e,e’).

This result once again showed that the serum extract from deer antler stem cells in anti-aging rat skin under the influence of UVA through stimulating the regeneration of rat skin structures at the microscopic level after 2 weeks of application.

### 3.7. Epidermal Thickness

**Figure 7:**
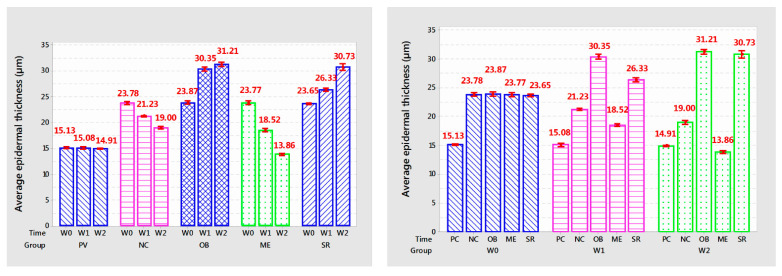
The graph shows the average epidermal thickness of the mouse skin after each week of treatment.

The epidermal thickness is shown in Table 3 and Figure 7.

Group PC: epidermal thickness tended to decrease slightly compared to the baseline time through each survey week, but this reduction was not statistically significant (*p* > 0.05); NC group: after stopping UVA irradiation for 2 weeks, the epidermal thickness decreased sharply but was still higher and different from the PC treatment (*p* < 0.01). In the ME group, the epidermal thickness tended to decrease sharply compared to before application (5.25 µm after 1 week of application and 4.66 µm after 2 weeks of application, *p* < 0.001). Two groups of OB and SR: Epidermal thickness of the OB treatment increased rapidly after 1 week of application (6.48 µm; *p* < 0.001) but increased slowly at the 2nd week of application. After 2 weeks of application, both treatments have similar epidermal thickness (*p* > 0.05), the graph tends to be horizontal, it can be seen that the epidermal thickness increases slowly and gradually stably. This is a positive sign for the skin, the increased thickness of the epidermis helps strengthen the skin barrier to resist external damage. The support for epidermal regeneration of the stem cell extract in the SR treatment showed that the epidermis had more positive expression than the OB treatment.

This may be closely related to the ingredient in SR, which is coconut oil, which has the ability to increase the thickness of the epidermis to protect against damage from UVA rays. Through the tanning pattern, the epidermis in the OB treatment tended to be thickened but accompanied by a large amount of keratinized cells on the outside (Figure 6c,c’). In contrast, when using SR, the epidermis was relatively thickened, even thinner than in the OB treatment, besides, the surface of the epidermis applied with SR had very few keratinocytes (Figure 6e,e’). We conclude that the incorporation of stem cell extract in SR promotes the regeneration and proliferation of the epidermis, which strengthens the skin’s barrier under the action of coconut oil and reduces the amount of keratinized cells on the surface of the skin. face, this is a beneficial reaction for the skin.

### 3.8. Collagen Density 

**Figure 8:**
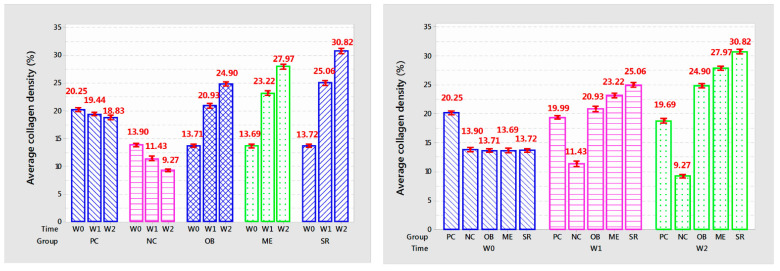
The graph shows the average collagen density (%) of the mouse skin after each week of treatment.

The results of collagen density after each week of applying the experimental product are shown in Table 4 and Figure 8.

The results showed that the collagen density per week of applying the product in each group was different with very high confidence (*p* < 0.001). Collagen density in PC and NC mice gradually decreased with each week (*p* < 0.001). In the OB group, collagen density increased and was significantly different after 2 weeks of application (*p* < 0.001), increasing from 13.71% to 20.93% after 1 week and 24.9% after 2 weeks. In addition, after 1 week of application, the results showed the similarity between the OB treatment and the PC treatment at the same time of the survey (*p* > 0.05) and the high difference at the 2nd week (*p* < 0.001). In the ME group, collagen density increased rapidly after 1 week of application (from 13.69% to 23.22%, *p* < 0.001) and increased slowly at week 2 (from 23.22% to 27.97%, *p* < 0.001). In particular, the SR group showed the highest efficiency with a high increase in collagen density separate from the other groups after each week of applying the product, after the week it increased from 13.72% to 25.06% and 30.82%, (*p* < 0.001). Compared with control, collagen density increased about 28.9% after 1 week of application and 63.76% after 2 weeks. Results of measuring collagen density showed that deer antler stem cell extract serum initially showed effectiveness in anti-aging mouse skin under the influence of UVA rays through stimulating collagen deposition after 2 weeks of use.

Overall, from the results obtained, it was found that the SR group gave outstanding anti-aging effects, helping to reduce the number of wrinkles, increase skin elasticity as well as stimulate collagen and epidermal proliferation.

## 4. Discussion

With our formulation process, the serum containing 0.01–2.5% extract is added to the foundation, making the serum product highly stable and helping to improve skin aging significantly after 2 weeks of use. Skin that has been applied with the serum product shows a smooth and elastic skin surface with very few fine lines and wrinkles. Serum reduces the number of wrinkles (48.09% compared to commercial serum (ME) and 60.31% compared to control (PC)), reduces skin recovery time (39.31% compared to ME and 67.1% compared to PC) after two weeks of use. Skin that has been applied with the serum product shows a smooth and elastic skin surface with very few fine lines and wrinkles. Serum reduces the number of wrinkles (48.09% compared to commercial serum (ME) and 60.31% compared to control (PC)), reduces skin recovery time (39.31% compared to ME and 67.1% compared to PC) after two weeks of use. Besides, collagen density increased 10.18% compared to ME and 63.76% compared to PC. Epidermal thickness increased by 106.1 % compared to PC and 121.7% compared to ME. On the other hand, base coconut oil also gave positive results: increased collagen density 7.6% compared to PC after 1 week of use and 32.23% after 2 weeks, epidermal thickness increased 101–109.32% after 1 and 2 weeks. In addition, compared with PC, the number of wrinkles was reduced by 7.4% after 2 weeks of use, and the skin recovery time was reduced by 37%.

Coconut oil base (group OB) with ingredients containing VCO with characteristics suitable to be an ingredient in anti-aging cosmetics, coconut oil consists of 90–95% saturated triglycerides with moisturizing effect the skin as an emollient by smoothing the edges and filling in the gaps between the cells [36,38]. The triglycerides in coconut oil are broken down by lipases in the normal flora of the skin into glycerin and fatty acids [7]. Glycerin is a strong humectant that attracts water to the epidermis from the outer environment and deeper skin layers [38]. Thanks to this feature, coconut oil helps to improve skin dryness caused by UVA while supporting the improvement of elasticity as well as reducing wrinkles on the skin.

The results showed that the loss of collagen when using coconut oil increased from 13.71% to 20.93 and 24.9%, it can be seen that VCO has the ability to stimulate and promote collagen deposition. VCO contains high levels of ferulic acid and p-coumaric acid which have antioxidant properties [18], this contributes to the reduction of ROS and limits the activation of MMPs in the skin. There is ample evidence to support VCO’s ability to promote wound healing through the promotion of epithelial regeneration and collagen synthesis [21]. There was an increased level of pepsin-soluble collagen (higher collagen cross-linking) in VCO treated wounds compared to controls. Histopathology showed increased fibroblast proliferation and neovascularization in these wounds [39]. 

UVA induces an inflammatory response and increases intracellular ROS production leading to the induction and activation of MMPs leading to collagen depletion. VCO-supplemented skin showed an inhibitory effect on mRNA expression and production of TNF-α (important initiator of inflammatory cytokines) [40]. Thereby limiting inflammation and ROS formation leading to non-expression of MMPs, helping collagen in the skin to be proliferated again.

The combination of base coconut oil and deer antler stem cell extract showed the highest anti-aging effect with noticeable changes in the skin’s surface as well as parameters of wrinkles, elasticity and collagen density. After 2 weeks of using SR, there was a significant reduction in the number and depth of wrinkles, the time for skin to recover was also significantly reduced (0.46–0.71 s) and collagen density increased by about 67.1% compared to PC and completely different from other treatments, even decreased compared to ME during the same survey period. It is possible to notice that the elasticity of the skin is also improved and enhanced after 2 weeks of use. 

The results of using the extract showed that the activity of secretions from deer antler stem cells also brought positive effects on the microenvironment of the host tissue, even promoting cell migration and increasing the ability to wound healing of fibroblasts in vitro and in vivo [28]. These secretions have regenerative effects such as basic fibroblast growth factor (bFGF), endothelial growth factors (VEGFs), platelet-derived growth factors (PDGFs), transforming growth factor—beta 2 (TGF-β2), which serve as endocrine factors for cell-to-cell communication, and vesicles from deer antler stem cells act as endocrine mediators to regulate cellular responses [28].

Among them, VEGF and TGF-β are thought to play an important role in skin repair [41]. The promotion of TGF-β, VEGF, and bFGF production further enhances angiogenesis and promotes cell proliferation and migration that support changes in the extracellular matrix (ECM) during the proliferative phase [42].

TGF-β stimulates the transcription of collagen and other genes encoding extracellular matrix proteins in vitro and in vivo and plays a central role in tissue repair and pathological fibrosis [43]. In addition, TGF-β stimulates cells to increase protein synthesis from the ECM, and simultaneously reduces collagenase [44]. With a high content of TGF-β2 in the extract, it supports the production and regeneration of collagen as well as protein synthesis for ECM, which contributes to improving elasticity as well as reducing wrinkles on the skin. The formation of skin structure requires removal of old tissue and revascularization. A framework of collagen and elastin fibers replaces old ones through tissue remodeling involving TGF-β-mediated synthesis of new collagen and breakdown of old collagen by PDGF [45]. 

The combination of coconut oil base and deer antler stem cell extract has shown anti-aging effects when promoting the advantages of coconut oil such as anti-inflammatory, antioxidant and moisturizing factors and growth factors in extracts such as bFGF, VEGFs, PDGFs, TGF-β2 promote skin healing, increase new collagen synthesis, angiogenesis and proliferation of new cells.

The increase in epidermal thickness with the use of coconut oil is seen as a response to the enhancement of the skin barrier. Keratinocyte differentiation and skin barrier function are enhanced by VCO through the promotion of genes encoding proteins present in the top layer of the epidermis, including involucrin (IVL), filaggrin (FLG), aquaporin (AQP-3) [19]. Among them, AQP-3 is found in the basal cells of the epidermis, is a humectant that also plays an important role in maintaining osmotic pressure through the layers of the skin [46]. VCO induction facilitates the functions of AQP-3 allowing for better water distribution and retention, glycerol helps to moisturize the skin [19]. 

Filaggrin is an essential protein required for epidermal growth and its intracellular metabolism leads to hydration of the stratum corneum and physiological pH balance [47]. Human involucrin is an important precursor for cell envelope and binding formation that acts as a major substrate for enzymes [48]. VCO increases filaggrin, involucrin levels and regulates mRNA expression levels in HaCaT cells and thus it can promote cell envelope formation and binding [19]. 

An advantage found from the epidermis in the skin of mice using serum from coconut oil combined with TBG extract (treatment SR) is that the link between the epidermis and dermis is somewhat tighter, which also contributes to improved improves skin texture and adds stability to the skin. On the other hand, the epidermis when using SR was relatively thickened, even thinner than in the OB treatment, besides, the surface of the epidermis applied with SR had very few keratinocytes (Figure 6e,e’) while that the epidermis in the OB treatment tended to thicken but accompanied by a large amount of keratinized cells on the outside (Figure 6c,c’). We hypothesized that the combination of TBG extract in SR helps to control the excessive proliferation of the epidermis under the effect of coconut oil while supporting and stimulating the regeneration of the epidermis, reducing the amount of keratinized cells on the skin surface. This mechanism needs more evidence to be conclusive.

## 5. Conclusions

In summary, coconut oil has demonstrated many advantages to meet the requirements for the composition of an anti-aging cosmetic product with the ability to moisturize, antioxidant, strengthen the skin barrier and support regeneration collagen in ECM. The combination of stem cell extract and coconut oil helps to promote the restructuring of ECM and improve the condition of aging skin by stimulating angiogenesis, promoting cell proliferation, stimulating collagen deposition and other genes encoding extracellular matrix proteins, thereby increasing skin elasticity, reducing wrinkles and strengthening the skin barrier.

Serum based on coconut oil combined with TBG deer antler extract showed significant improvement in aging skin conditions: reducing the number of wrinkles and the recovery time of the skin; increase the cohesion between the epidermis and the dermis and increase collagen density in a short time.

## Figures and Tables

**Table 1:** Average number of wrinkles after each week of treatment.

	**Time**	W0	W1	W2
Group				
**PC**		5.30 ± 0.55 ^Aa^	6.39 ± 0.64 ^A^^b^	7.56 ± 0.91 ^B^^b^
**NC**		8.67 ± 0.45 ^B^^a^	9.94 ± 0.55 ^B^^b^	11.33 ± 0.77 ^Ac^
**OB**		8.70 ± 0.40 ^B^^a^	8.17 ± 0.49 ^C^^a^	7.00 ± 0.69 ^BCb^
**ME**		8.78 ± 0.50 ^B^^a^	7.50 ± 0.61 ^Cb^	5.78 ± 0.86 ^Cc^
**SR**		8.89 ± 0.50 ^B^^a^	6.06 ± 0.62 ^Ab^	3.00 ± 0.87 ^D^^c^

A, B, C, D represents the column difference at the 95% confidence level. a, b, c represent the row difference at the 95% confidence level.

**Table 2:** Average skin recovery time after each week of treatment.

	**Time**	W0	W1	W2
Group				
**PC**		1.22 ± 0.14 ^Aa^	1.72 ± 0.17 ^BCb^	2.16 ± 0.24 ^A^^c^
**NC**		2.60 ± 0.12 ^Ba^	3.02 ± 0.15 ^Ab^	3.50 ± 0.21 ^Bc^
**OB**		2.62 ± 0.08 ^Ba^	1.81 ± 0.10 ^Bb^	1.42 ± 0.14 ^Cc^
**ME**		2.63 ± 0.07 ^Ba^	1.66 ± 0.09 ^BCb^	1.17 ± 0.13 ^Dc^
**SR**		2.66 ± 0.11 ^Ba^	1.48 ± 0.14 ^Cb^	0.71 ± 0.19 ^Ec^

A, B, C, D, E represents the column difference at the 95% confidence level. a, b, c represent the row difference at the 95% confidence level.

**Table 3:** Average epidermal thickness of rat skin after each week of treatment.

	**Time**	W0	W1	W2
Group				
**PC**		15.13 ± 0.16 ^A^^a^	15.08 ± 0.27 ^A^^a^	14.91 ± 0.14 ^A^^a^
**NC**		23.78 ± 0.30 ^B^^a^	21.23 ± 0.17 ^B^^b^	19.00 ± 0.31 ^Bc^
**OB**		23.87 ± 0.33 ^Ba^	30.35 ± 0.42 ^Cb^	31.21 ± 0.41 ^Cc^
**ME**		23.77 ± 0.38 ^Ba^	18.52 ± 0.28 ^Db^	13.86 ± 0.20 ^Dc^
**SR**		23.65 ± 0.20 ^Ba^	26.33 ± 0.34 ^Eb^	30.73 ± 0.64 ^Cc^

A, B, C, D, E represents the column difference at the 95% confidence level. a, b, c represent the row difference at the 95% confidence level.

**Table 4:** Average collagen density (%) after each week of treatment.

	**Time**	W0	W1	W2
Group				
**PC**		20.25 ± 0.34 ^A^^a^	19.44 ± 0.31 ^A^^b^	18.83 ± 0.43 ^A^^c^
**NC**		13.90 ± 0.32 ^B^^a^	11.43 ± 0.45 ^B^^b^	9.27 ± 0.26 ^Bc^
**OB**		13.71 ± 0.30 ^Ba^	20.93 ± 0.46 ^Cb^	24.90 ± 0.35 ^Cc^
**ME**		13.69 ± 0.37 ^Ba^	23.22 ± 0.44 ^Db^	27.97 ± 0.39 ^Dc^
**SR**		13.72 ± 0.28 ^Ba^	25.06 ± 0.45 ^Eb^	30.82 ± 0.44 ^Cc^

A, B, C, D, E represents the column difference at the 95% confidence level. a, b, c represent the row difference at the 95% confidence level.

## Data Availability

Not applicable.
